# Effects of l-Arginine Plus Vitamin C Supplementation on Physical Performance, Endothelial Function, and Persistent Fatigue in Adults with Long COVID: A Single-Blind Randomized Controlled Trial

**DOI:** 10.3390/nu14234984

**Published:** 2022-11-23

**Authors:** Matteo Tosato, Riccardo Calvani, Anna Picca, Francesca Ciciarello, Vincenzo Galluzzo, Hélio José Coelho-Júnior, Angela Di Giorgio, Clara Di Mario, Jacopo Gervasoni, Elisa Gremese, Paolo Maria Leone, Antonio Nesci, Anna Maria Paglionico, Angelo Santoliquido, Luca Santoro, Lavinia Santucci, Barbara Tolusso, Andrea Urbani, Federico Marini, Emanuele Marzetti, Francesco Landi

**Affiliations:** 1 Fondazione Policlinico Universitario A. Gemelli IRCCS, 00168 Rome, Italy; 2 Department of Medicine and Surgery, LUM University, 70010 Casamassima, Italy; 3 Department of Geriatrics and Orthopedics, Università Cattolica del Sacro Cuore, 00168 Rome, Italy; 4 Immunology Core Facility, Gemelli Science Technological Park (GSTeP), Fondazione Policlinico Universitario A. Gemelli IRCCS, 00168 Rome, Italy; 5 Department of Cardiovascular Sciences, Università Cattolica del Sacro Cuore, 00168 Rome, Italy; 6 Metabolomics Research Core Facility, Gemelli Science and Technology Park (GSTeP), Fondazione Policlinico Universitario A. Gemelli IRCCS, 00168 Rome, Italy; 7 Department of Biochemistry and Clinical Biochemistry, Università Cattolica del Sacro Cuore, 00168 Rome, Italy; 8 Department of Chemistry, Sapienza University of Rome, 00185 Rome, Italy

**Keywords:** post-acute COVID-19 syndrome, SARS-CoV-2, 6 min walk test, handgrip strength, flow-mediated dilation, nitric oxide, nutraceuticals, oral supplement, persistent symptoms

## Abstract

Long COVID, a condition characterized by symptom and/or sign persistence following an acute COVID-19 episode, is associated with reduced physical performance and endothelial dysfunction. Supplementation of l-arginine may improve endothelial and muscle function by stimulating nitric oxide synthesis. A single-blind randomized, placebo-controlled trial was conducted in adults aged between 20 and 60 years with persistent fatigue attending a post-acute COVID-19 outpatient clinic. Participants were randomized 1:1 to receive twice-daily orally either a combination of 1.66 g l-arginine plus 500 mg liposomal vitamin C or a placebo for 28 days. The primary outcome was the distance walked on the 6 min walk test. Secondary outcomes were handgrip strength, flow-mediated dilation, and fatigue persistence. Fifty participants were randomized to receive either l-arginine plus vitamin C or a placebo. Forty-six participants (median (interquartile range) age 51 (14), 30 [65%] women), 23 per group, received the intervention to which they were allocated and completed the study. At 28 days, l-arginine plus vitamin C increased the 6 min walk distance (+30 (40.5) m; placebo: +0 (75) m, *p* = 0.001) and induced a greater improvement in handgrip strength (+3.4 (7.5) kg) compared with the placebo (+1 (6.6) kg, *p* = 0.03). The flow-mediated dilation was greater in the active group than in the placebo (14.3% (7.3) vs. 9.4% (5.8), *p* = 0.03). At 28 days, fatigue was reported by two participants in the active group (8.7%) and 21 in the placebo group (80.1%; *p* < 0.0001). l-arginine plus vitamin C supplementation improved walking performance, muscle strength, endothelial function, and fatigue in adults with long COVID. This supplement may, therefore, be considered to restore physical performance and relieve persistent symptoms in this patient population.

## 1. Introduction

A large share of COVID-19 survivors reports long-lasting clinical sequelae weeks or months after symptom onset: a condition known as post-acute COVID-19 syndrome or long COVID [1,2]. Long COVID encompasses a constellation of respiratory, cardiovascular, gastrointestinal, and neurological signs and symptoms, such as dyspnea, fatigue, dysrhythmias, heartburn, and memory and attention difficulties (“brain fog”), with a substantial impact on quality of life [3,4]. Long COVID is also associated with reduced physical function, which may hamper the complete resumption of pre-infection daily activities [5,6]. Several processes are currently being investigated for their involvement in the pathophysiology of long COVID, including viral persistence, chronic inflammation, autoimmunity, perturbation in metabolic and redox homeostasis, and endothelial dysfunction [7,8]. The heterogeneity of clinical manifestations of long COVID has hampered the devising of specific treatments for the condition, such that its management is mostly based on symptomatic treatments and healthy lifestyle recommendations.

Several nutritional supplements and bioactive foods are being investigated to counteract long COVID [9]. l-arginine is a key regulator of immune, respiratory, and endothelial function [10,11]. Its pleiotropic properties are regulated by two main metabolizing enzymes, nitric oxide (NO) synthase and arginase [12]. The flux of l-arginine towards NO synthesis is associated with beneficial effects on immune and vascular health, while its catabolism to ornithine by arginase has been associated with an abnormal immune response and endothelial dysfunction [13,14]. Accumulating evidence indicates that l-arginine metabolism is altered in patients with COVID-19 [10,11]. During acute COVID-19, the upregulation of arginase activity reduces the circulating levels of l-arginine and shifts its metabolism away from NO production to induce immune and endothelial dysfunction, inflammation, and thrombosis, which ultimately lead to vascular occlusion and multiorgan failure [11]. Indeed, lower plasma levels of l-arginine and higher arginase activity have been found both in patients with COVID-19 and in children with the multisystem inflammatory syndrome (MIS-C) compared with healthy controls [15]. A decrease in plasma l-arginine levels has also been described in acute COVID-19 and is associated with the expansion of myeloid-derived suppressor cells and impaired T-cell proliferation, two cardinal inflammatory features of severe disease [16,17].

Based on these observations, strategies to restore circulating levels of l-arginine and increase NO bioavailability have been proposed to counteract immune, respiratory, and vascular complications of COVID-19 [11,18]. Vitamin C may support the beneficial effects of l-arginine on endothelial function by increasing intracellular tetrahydrobiopterin, a co-factor needed for the oxidation of l-arginine to NO, in endothelial cells [12,19]. In vitro, l-arginine supplementation restores the proliferative capacity of T-cells obtained from patients with acute respiratory distress syndrome during COVID-19 [17]. Furthermore, oral l-arginine supplementation has been shown to reduce the need for respiratory support and the length of hospital stay in patients with severe COVID-19 [20]. Finally, oral supplementation with l-arginine plus vitamin C reduced the burden of persisting symptoms and ameliorated perceived exertion in a large cohort of patients with long COVID [21].

Previous studies have shown that l-arginine supplementation improves respiratory function and exercise tolerance in patients with pulmonary diseases [22] and in those with congestive heart failure [23] as well as in heart transplant recipients [24]. In addition, supplementation with l-arginine may increase aerobic and anaerobic performance in healthy adults, especially in untrained individuals [25,26]. However, other studies found no effects of l-arginine supplementation on human performance [27,28].

To further explore the potential benefits of l-arginine supplementation on COVID-19 outcomes, we conducted a single-blind randomized controlled trial to assess the effects of a 28-day oral supplementation with l-arginine plus vitamin C on physical performance, muscle strength, endothelial function, fatigue persistence, and systemic l-arginine bioavailability in adults with long COVID. 

## 2. Materials and Methods

### 2.1. Study Design and Participants

This was a single-center, single-blind, placebo-controlled randomized clinical trial that was conducted at the post-acute COVID-19 outpatient clinic of the Fondazione Policlinico A. Gemelli IRCCS (Rome, Italy) [29] from 1 July 2021 to 30 April 30, 2022. The study protocol was approved by the institutional ethics committee (prot. no. 0013008/20). Written informed consent was collected from all participants prior to enrolment. The trial was conducted in accordance with the guidelines of the International Council for Harmonization of Technical Requirements for Pharmaceuticals for Human Use Good Clinical Practice and the principles of the Declaration of Helsinki. The trial was registered on ClinicalTrials.gov (NCT04947488).

Eligible participants were men and women aged between 20 and 60 years who met the following criteria: (a) previous confirmed SARS-CoV-2 infection, certified by a positive RT–PCR molecular swab test; (b) a negative COVID-19 swab test at least four weeks prior to the start of trial operations; (c) long COVID diagnosis according to the World Health Organization criteria [30]; and (d) persistent fatigue, operationalized as the response “most or all the time” to item seven of the Center for Epidemiological Studies Depression Scale (“I felt that everything I did was an effort”) [31]. The main exclusion criteria were intolerance to either supplement ingredient (i.e., l-arginine and vitamin C), conditions and/or therapies that may interfere with trial outcomes and procedures (e.g., pregnancy or breastfeeding, chronic pulmonary disease, diabetes, use of antihypertensive drugs, steroids, or non-steroidal anti-inflammatory drugs, immunosuppressants, nitrates), and engagement in other intervention trials for long COVID.

Eligible participants were randomized using a random number generator in a 1:1 ratio to receive a twice-daily oral supplementation with either a combination of 1.66 g l-arginine plus 500 mg liposomal vitamin C (Bioarginina^®^ C, Farmaceutici Damor, Naples, Italy) or a placebo for 28 days. Vials containing the active supplement or the placebo were supplied by Farmaceutici Damor and were made to be indistinguishable in appearance. 

The primary endpoint was the change from baseline to day 28 in the distance walked on the 6 min walk test. The secondary endpoints were changes from baseline to day 28 in handgrip strength and flow-mediated dilation, and fatigue persistence through day 28. Serum l-arginine levels were measured before the intervention and at 28 days. Participants were asked to refrain from exercising and consuming any vasoactive products (e.g., tobacco, caffeinated drinks) for at least 12 h prior to the assessment of physical performance, muscle strength, endothelial function, and blood draw. The last consumption of the supplements occurred the evening before the tests. Outcome assessors were unaware of the group assignment. Adverse events were recorded and compared between the intervention groups.

### 2.2. Anthropometric and Clinical Data

Body height and weight were measured through a standard stadiometer and an analog medical scale, respectively. Body mass index (BMI, kg/m^2^) was calculated as the ratio between the weight and the square of height. The severity of the acute COVID-19 episode was categorized as follows: (a) no hospitalization, (b) hospitalization, and (c) intensive care unit (ICU) admission. The time from COVID-19 diagnosis to the study inclusion was calculated based on self-report.

### 2.3. Measurement of Serum l-Arginine Concentration

Blood samples were collected at the baseline and after 28 days of intervention. Blood was drawn after overnight fasting using standard collection tubes (BD Vacutainer^®^; Becton, Dickinson and Co., Franklin Lakes, NJ, USA). Samples were left at room temperature for 30 min and were then centrifuged at 1000× *g* for 10 min at 4 °C. Serum aliquots were stored at −80 °C until analysis. Serum samples from 20 age- and sex-matched blood donors without evidence of previous SARS-CoV-2 infection were collected and used as a “healthy” reference. Serum l-arginine levels were determined using an in-house validated liquid chromatography with tandem mass spectrometry method [32]. The chromatographic separation was performed with an ACQUITY UPLC I-Class System (Waters, Milford, MA, USA) using a HILIC column. Analyte detection was accomplished with a triple quadrupole Xevo-TQs Micro (Waters) equipped with an electrospray ion source operating in positive ion mode. A multiple reaction monitoring experiment was optimized for the detection and quantification of l-arginine.

### 2.4. Primary Outcome

The primary outcome measure was the distance walked on the 6 min walk test [33]. The test is a valid and easy-to-perform measure of exercise capacity in people with chronic lung disease and is increasingly being used to assess the sequelae of COVID-19 [34]. All participants completed the test at baseline and after 28 days of intervention. The test was performed on a 20 m-long indoor hallway, with markers placed at each end of the track, as previously described [35]. The total distance walked in 6 min was recorded in meters.

### 2.5. Secondary Outcomes

All secondary outcome measures were collected at baseline and after 28 days of intervention.

Muscle strength was assessed by handgrip strength testing [36] using a hydraulic hand-held dynamometer (North Coast Medical, Inc., Morgan Hill, CA, USA) according to international standard protocols, as detailed elsewhere [37]. Briefly, the participant was requested to sit on a standard chair, with the elbow near the trunk and bent at 90°, the hand in a neutral position, with the thumb pointing upwards. The measure was obtained after participants performed one familiarization trial with both hands. The highest reading (reported in kg) out of three trials was used for the analysis. Handgrip strength values at the baseline and post-intervention were compared with age- and sex-specific reference values [37].

The endothelial function was assessed non-invasively by measuring the brachial artery dilation following a transient period of forearm ischemia (flow-mediated dilation test) [38]. Flow-mediated dilation was measured by Doppler ultrasonography, using an iU22 2D ultrasound system (Philips Electronics, Amsterdam, The Netherlands) according to standard protocols [38,39], as described elsewhere [40]. In brief, the diameter of the brachial artery was measured at baseline and after the abrupt release of a blood pressure cuff that arrested the forearm circulation (by applying a pressure of 250 mmHg for 5 min). Flow-mediated dilation, which is mainly mediated by NO, was expressed as the percent increase in the arterial diameter following cuff release compared with the baseline diameter.

The persistence of fatigue was operationalized as the response “most or all the time” to item seven of the Center for Epidemiological Studies Depression Scale (CES-D, “I felt that everything I did was an effort”) [31]. This operationalization of fatigue is commonly used in studies on physical frailty [41]. Furthermore, item seven of the CES-D was shown to be more related to fatigue than to depression [42].

### 2.6. Statistical Analysis

For the estimation of the sample size, we used reference values for the 6 min walk test in healthy adults published by Chetta et al. [43]. A sample size of 42 participants, 21 per intervention arm, was estimated to provide 80% power to detect a between-group difference of at least 35 m on the 6 min walk test, considering a standard deviation of 50 m and an alpha level of 5%. The recruitment target was increased to 50 participants (25 per group) to account for a 20% dropout rate. The 35 m cut-point was chosen as it corresponds to the minimal clinically important difference for the test [44].

The normal distribution of data were assessed via the Shapiro−Wilk test. Anthropometric, clinical, and functional characteristics of the study participants were reported as the median (interquartile range, IQR) for continuous variables and as absolute values (percentages) for categorical variables. Changes from baseline for continuous variables were expressed as deltas (values at 28 days minus the values at baseline), and differences between the intervention groups were evaluated using the Student’s *t*-test for normally distributed variables or the Mann–Whitney U test for skewed variables. Mean differences and effect size values (Cohen’s d for Student’s *t*-test and rank biserial correlation for Mann−Whitney U test) were reported. Chi-squared or Fisher tests were used to assess differences between groups in categorical variables. Analyses of intervention effects were based on the intention-to-treat principle. All tests were two-sided with a statistical significance set at *p* < 0.05. All analyses were performed using Jamovi freeware version 2.0.0.0 (The Jamovi project, 2021; retrieved from https://www.jamovi.org; accessed on 19 July 2021).

Multivariate classification models, based on partial least squares discriminant analysis (PLS–DA) [45], were built and validated by double cross-validation [46] to gain a more comprehensive appraisal of the effects of interventions on the variables of interest. The potential influence of confounding factors (i.e., age and sex) was also evaluated. Multivariate analyses were performed using in-house routines running under the MATLAB R2015b environment (The MathWorks, Natick, MA, USA) and are detailed in Supplementary Material.

## 3. Results

**Figure 1:**
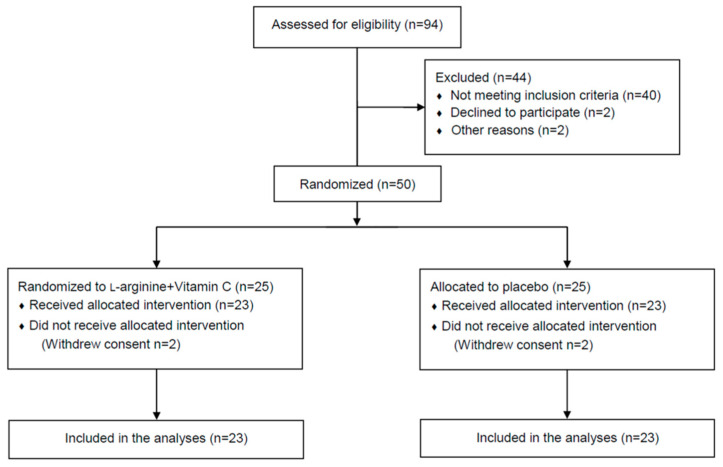
CONSORT diagram of participant flow through the study.

Out of 94 candidate participants screened, 50 (53.2%) met the inclusion criteria and agreed to be randomized to receive either the l-arginine plus vitamin C (*n* = 25) or placebo (*n* = 25) intervention. Two participants in each group withdrew their consent before receiving the allocated intervention and were not included in the analysis (Figure 1).

The anthropometric, clinical, and functional characteristics of the study participants at baseline were comparable between the intervention groups (Table 1).

**Figure 2:**
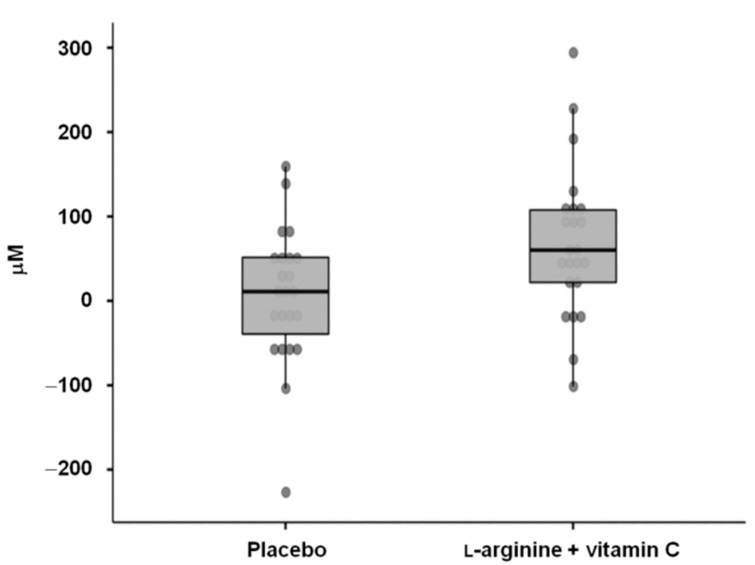
Changes from baseline to day 28 in serum l-arginine levels in the two intervention groups.

Participants had a median (IQR) age of 50.5 (14.0) years and were predominantly women (65.2%). Approximately half of the participants needed hospitalization during the acute COVID-19 episode, and four (8.7%) were admitted to ICU. The median (IQR) time that elapsed from COVID-19 diagnosis to inclusion in the study was 254.0 (136.5) days. The median (IQR) distance walked on the 6 min walk test was 520 (90) m, while the handgrip strength was 22.6 (14.4) kg. Flow-mediated dilation at baseline was 9.8%. The median (IQR) serum l-arginine concentration was 170.6 (88.0) µM, with no differences between the intervention groups. However, serum l-arginine values were lower than those observed in the sample without evidence of previous SARS-CoV-2 infection (median (IQR) 222.1 (23.1) µM; *p* = 0.04). At 28 days, serum l-arginine concentrations increased more in the participants who received l-arginine plus vitamin C supplementation (+60.2 (85.8) µM) than in the placebo group (+11.0 (90.8) µM; *p* = 0.02; mean difference 62.4 µM, 95% confidence interval (CI): 11.1–113.7 µM; effect size = 0.72) (Figure 2). After 28 days of l-arginine plus vitamin C supplementation, serum l-arginine levels in the active group (222.8 (88.6) µM) were comparable to those of the controls with no previous SARS-CoV-2 infection (*p* = 0.8).

l-arginine plus vitamin C supplementation was safe and well tolerated, and no adverse events were recorded.

### Efficacy Endpoints

**Figure 3:**
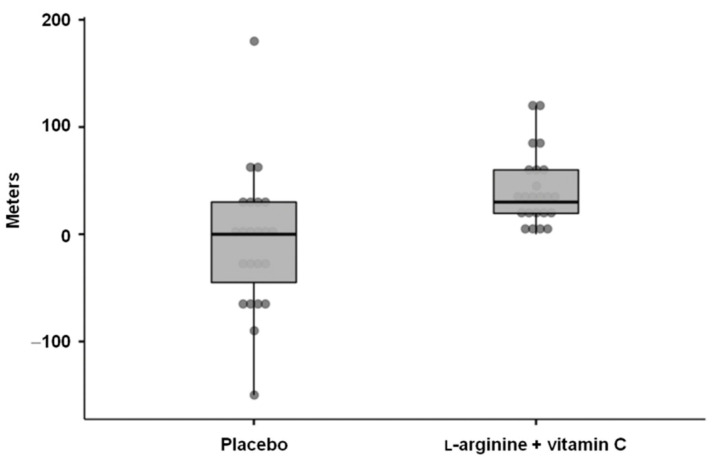
Changes from baseline to day 28 in the 6 min walk test distance in the two intervention groups.

l-arginine plus vitamin C significantly increased the distance walked on the 6 min walk test (median (IQR) change from baseline: +30.0 (40.5) m) compared with the placebo (+0.0 (75.0) m; *p* = 0.001; mean difference = 50 m, 95% CI: 20.0–80.0 m; effect size = 0.56) (Figure 3). 

**Figure 4:**
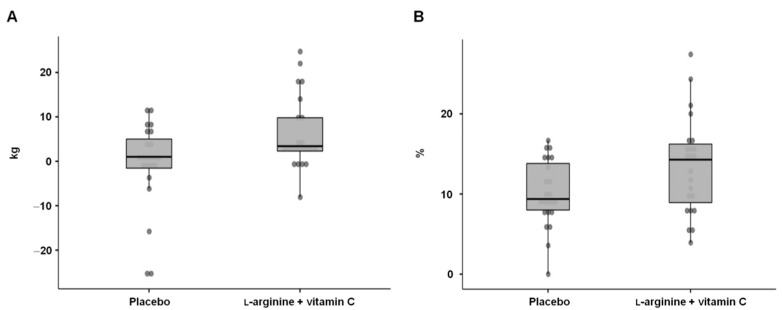
Changes from baseline to day 28 in (**A**) handgrip strength and (**B**) flow-mediated dilation in the two intervention groups.

At 28 days, l-arginine plus vitamin C supplementation induced greater improvements in handgrip strength (+3.4 (7.5) kg) compared with the placebo (+1.0 (6.6) kg, *p* = 0.03; mean difference = 3.4 kg, 95% CI: 0.5–9.4 kg; effect size = 0.37) (Figure 4A). At baseline, around 60% of the study participants had a handgrip strength below the 25th percentile of age- and sex-specific reference values [37]. After 28 days of intervention, more participants who received l-arginine plus vitamin C than those in the placebo group (57% vs. 30%) were above the first quartile of handgrip strength reference values [37], although the difference between the groups did not reach the statistical significance (*p* = 0.07). 

Flow-mediated dilation was greater in participants who received l-arginine plus vitamin C compared with the placebo (14.3% (7.3) vs. 9.4% (5.8), *p* = 0.03; mean difference = 3.4%; 95% CI: 0.4–6.5; effect size = 0.66) (Figure 4B). 

Finally, on day 28, fatigue was reported by two participants (8.7%) in the l-arginine plus vitamin C supplementation group and 21 (80.1%) in the placebo group (*p* < 0.0001).

As described in Supplementary Material S1, the multivariate classification showed a high degree of correlation among the four variables of interest (i.e., 6 min walk distance, handgrip strength, flow-mediated dilation, and serum l-arginine concentration). The average classification accuracy of the participants in the two intervention arms was 77.7 ± 1.9% (*p* < 0.001). Participant classification was unaffected by age. Treatment effects were more evident in women than men. This may be due to the low number of male participants, most of whom were, however, correctly classified in the full model.

## 4. Discussion

In the present clinical trial, we showed that the l-arginine plus vitamin C supplementation improved walking performance, muscle strength, and endothelial function, reduced fatigue and restored serum l-arginine concentrations in adults with long COVID. These findings support the view that increasing NO bioavailability through the synergistic effects of l-arginine and vitamin C ameliorates post-acute COVID-19 sequelae [19].

The 6 min walking distance is a useful metrics of exercise capacity after COVID-19, as it correlates with the severity of acute disease and with pulmonary function/structure impairment in the post-acute phase [34,47]. In our study, participants allocated in the l-arginine plus vitamin C group showed clinically meaningful improvements [44] from baseline in the distance walked on the 6 min test compared with those who received a placebo. This finding aligns with previous evidence on the beneficial effects of l-arginine supplementation on pulmonary function and exercise capacity of patients with chronic lung diseases [22]. A short-term oral administration of l-arginine significantly decreased the mean pulmonary arterial pressure and vascular resistance and improved peak oxygen consumption and dead-space ventilation in patients with precapillary pulmonary hypertension [48]. A 12-week l-arginine supplementation combined with a home-based walking program increased the 6 min walking distance, peak aerobic capacity, and quality of life in clinically stable patients with pulmonary arterial hypertension [49]. Moreover, oral l-arginine supplementation boosted NO synthesis, improved endothelial function, and increased exercise tolerance in patients with congestive heart failure [50] and in heart transplant recipients [24].

A recent systematic review and meta-analysis on the effects of l-arginine supplementation on athletic performance indicated that either acute or chronic l-arginine supplementation could enhance both aerobic and anaerobic performance [25]. Based on these findings, the authors concluded that l-arginine supplementation with 1.5–2 g daily from four to seven weeks and 10–12 g daily for eight weeks could be recommended to improve aerobic and anaerobic performance, respectively [25]. Interestingly, untrained or moderately trained individuals seem to obtain greater gains in exercise performance after l-arginine supplementation than those who are highly trained [26]. While no conclusive evidence exists on the beneficial effects of l-arginine supplementation on human performance, our findings indicate that a short course of l-arginine plus vitamin C supplementation may positively impact the exercise capacity of adults with long COVID. 

Handgrip strength is a valid indicator of general health and a powerful predictor of disability, morbidity, and mortality across all life stages [51,52,53,54]. During an acute COVID-19 episode, low handgrip strength has been associated with an increased risk of hospitalization and poor outcomes [55,56,57]. In adults who survived severe COVID-19, handgrip strength values after six months of hospital discharge were significantly lower than the healthy controls [58]. Moreover, in a cohort of 541 individuals who recovered from COVID-19, low handgrip strength values were associated with a higher number of persistent symptoms, including fatigue and dyspnea [59]. In our study, approximately 60% of participants had low handgrip strength at baseline. Those who received l-arginine plus vitamin C experienced a greater increase in handgrip strength than the participants in the placebo group after 28 days of intervention. Remarkably, at day 28, more than half of the participants randomized to l-arginine plus vitamin C had handgrip strength values above the 25th percentile of age- and sex-specific reference values [37], compared with 30% in the placebo group. Indeed, l-arginine supplementation (either alone or in combination with other amino acids and derivatives) is among the nutritional strategies proposed to preserve muscle mass and function/strength and manage sarcopenia in older adults [60,61].

Flow-mediated dilation is a non-invasive measure of endothelial function and vascular health [62]. Recent studies showed that patients with acute COVID-19 and convalescent survivors had reduced flow-mediated dilation values, which supports the central role of endothelial dysfunction throughout the disease course [40,63,64,65]. In the present investigation, l-arginine plus vitamin C supplementation induced a greater flow-mediated dilation than the placebo. Since flow-mediated dilation is, at least partly, mediated by NO bioavailability [66], our findings provide the first evidence that the combination of l-arginine plus vitamin C may be effective at improving endothelial function in post-acute COVID-19 through increasing NO synthesis. This view is in line with the results of a meta-analysis of randomized clinical trials showing that short-term oral l-arginine supplementation improves endothelial function in both healthy individuals and those with cardiovascular disease [67].

Fatigue is one of the most prevalent and burdensome symptoms in people with long COVID [2,68]. Of note, after 28 days of l-arginine plus vitamin C supplementation, only two participants reported fatigue compared with 21 who had received a placebo. This finding is in keeping with recent evidence from a large Italian multicenter clinical study of patients with long COVID, which reported an amelioration of fatigue and perceived exertion following 30 days of supplementation with l-arginine plus vitamin C [21]. Hence, the synergistic effects of l-arginine and vitamin C on NO synthesis may play a favorable role not only on the endothelial function, but also on immune response regulation, two major determinants of fatigue in long COVID and chronic fatigue syndromes [69,70,71].

Some limitations should be taken into account in the interpretation of the study results. The sample size was adequately powered for the primary outcome. However, owing to the small number of participants and the single-center nature of the study, our results should be considered preliminary. Further trials with larger populations, conducted in multiple centers, and using different study methodologies (e.g., longer intervention, crossover design) are warranted to confirm these promising findings. The levels of physical activity as well as dietary habits of study participants may have influenced the intervention effects. However, participants were requested to refrain from exercising and limit the ingestion of foods rich in arginine or with vasoactive properties for at least 12 h before study visits. Multivariate analyses suggested that the effects of l-arginine plus vitamin C supplementation were more evident in women. However, the study was not powered to evaluate sex-specific differences in the response to treatments. Because a data safety monitoring board was not appointed, we opted for a single-blind approach to maximize participant safety. As mentioned earlier, to preserve the trial integrity, all outcome measures were assessed by investigators who were blind to the participant group assignment. Although well-established physical performance and muscle strength measures were used, it is possible that more sophisticated aerobic and anaerobic tests might provide additional information on the effects of l-arginine plus vitamin C supplementation in adults with long COVID. Finally, while the evaluation of serum l-arginine levels provided relevant information on the effectiveness of the proposed intervention, we cannot exclude the possibility that a more comprehensive evaluation of l-arginine metabolism (e.g., measurement of citrulline, ornithine, and methyl-arginine compounds) may provide further insights into the mechanisms by which the beneficial effects of l-arginine and vitamin C supplementation on the outcomes of interest are conveyed.

## 5. Conclusions

l-arginine plus vitamin C supplementation improved exercise capacity, muscle strength, endothelial function, and fatigue in adults with long COVID. The combination of l-arginine plus vitamin C may therefore be proposed as a remedy to restore physical performance and relieve persistent symptoms in people with long COVID.

## Figures and Tables

**Table 1:** Baseline characteristics of study participants.

Characteristic	l-Arginine + Vitamin C (*n* = 23)	Placebo (*n* = 23)	Total (*n* = 46)
Age, years	50.0 (16.5)	51.0 (11.0)	50.5 (14.0)
Women, *n* (%)	15 (65.2)	15 (65.2)	30 (65.2)
BMI, kg/m^2^	24.8 (5.9)	25.5 (6.5)	25.0 (6.5)
Severity of acute COVID-19, *n* (%)			
No hospitalization	8 (34.8)	12 (52.2)	20 (43.5)
Hospitalization	13 (56.5)	9 (39.1)	22 (47.8)
ICU admission	2 (8.7)	2 (8.7)	4 (8.7)
Time from COVID-19 diagnosis, days	240.0 (118.5)	269.0 (127.0)	254.0 (136.5)
6 min walk test distance, m	520.0 (49.5)	540.0 (120.0)	520.0 (90.0)
Handgrip, kg	22.5 (16.0)	22.6 (12.3)	22.6 (14.4)
Flow-mediated dilation, %	10.5 (5.2)	8.9 (5.8)	9.8 (6.0)
Serum l-arginine, µM	167.2 (76.8)	175.0 (93.1)	170.6 (88.0)

Abbreviations: BMI, body mass index; ICU, intensive care unit. Data are expressed as median (interquartile range) for continuous variables and number (percent) for categorical variables.

## Data Availability

The data presented in this study are available from the corresponding author upon reasonable request pending approval by the Gemelli Against COVID Scientific Committee.

## Supplementary Materials

The following supporting information can be downloaded at: https://www.mdpi.com/article/10.3390/nu14234984/s1, Multivariate Classification by Partial Least Squares Discriminant Analysis.
