# Supplementary material for: Effects of l-Arginine Plus Vitamin C Supplementation on Physical Performance, Endothelial Function, and Persistent Fatigue in Adults with Long COVID: A Single-Blind Randomized Controlled Trial
Source: Nutrients. 2022 Nov 23;14(23):4984. doi: 10.3390/nu14234984 (PMC9738241; doi:10.3390/nu14234984)
Supplement: Supplementary file 1 [file nutrients-14-04984-s001.zip › nutrients-1941400-supplementary.pdf]

Multivariate Classification by Partial Least Squares Discriminant Analysis

1. Partial Least Squares Discriminant Analysis (PLS–DA)

Multivariate classification models were built using partial least squares discriminant analysis (PLS–DA) [45]. PLS–DA is a classification method that exploits the advantages of the PLS algorithm for dealing with correlated variables. The PLS algorithm was originally developed for regression problems and it relies on the projection of the predictor matrix X onto a reduced space of orthogonal latent variables, yielding a matrix of scores T (coordinates of the samples onto the latent variables subspace):

$$T = XR \qquad \text{(S1)}$$

R being a matrix of weights determining the projection.

A regression model is then established between the scores T and the response y to be predicted:

$$y = Tq \qquad \text{(S2)}$$

q being the regression coefficients.

The same approach can be used for classification by using a dummy binary y coding for class-belonging: the elements of y can be either 1 if the sample belongs to a category (here participants supplemented with L-arginine plus vitamin C) or 0 if it belongs to the other category (here, placebo).

Model validation was achieved through a double cross-validation (DCV) procedure [46]. DCV consists of two loops of cross-validation nested in one another: the outer loop mimics an external test set, while the inner loop is used for model selection (i.e., choosing the optimal number of latent variables). The procedure was repeated 50 times, changing the distribution of the samples in the different cancelation groups, and this made it possible to calculate confidence intervals for all the model parameters and the figures of merit.

To account for the repeated measure design of the study, the classification model was built using, for each participant, the difference between values at 28 days and values at baseline. Sex-specific models and models including age as a covariate were also built to evaluate the potential influence of these traditional confounding factors. Analyses were performed using in-house routines running under MATLAB R2015b environment (The MathWorks, Natick, MA, USA).

2. Results

To gain a more comprehensive appraisal of the effects of interventions on the variables of interest, data were processed through a PLS–DA-based approach. In order to obtain an unbiased validation of the results and estimate the confidence intervals of the main figures of merit, repeated DCV was conducted, with 10 and eight cancelation groups in the outer and inner loops, respectively, and permutation test with 1000 randomizations.

First, a classification model was built using only the four variables of interest (6-min walk distance, handgrip strength, flow-mediated dilation, and serum L-arginine concentrations). The optimal model complexity was found to be 1 ± 1 latent variables, confirming the high degree of correlation among the predictors. When considering the classification figures of merit (estimated on the outer loop, i.e., on samples in turn not included in the model used for their prediction), an average classification accuracy of 77.7 ± 1.9% was achieved, corresponding to 83.8 ± 3.0% and 71.6 ± 2.7% correct classification rate for the active group and placebo, respectively. The non-parametric estimation of the distribution of these figures of merit under the null hypothesis by permutation testing indicated that they were statistically significant ($p < 0.001$).

A graphical representation of the discriminant ability of the model is depicted in Figure S1, where the repeated DCV outer loop sample scores and the variable weights along the only canonical variate of the model are displayed.

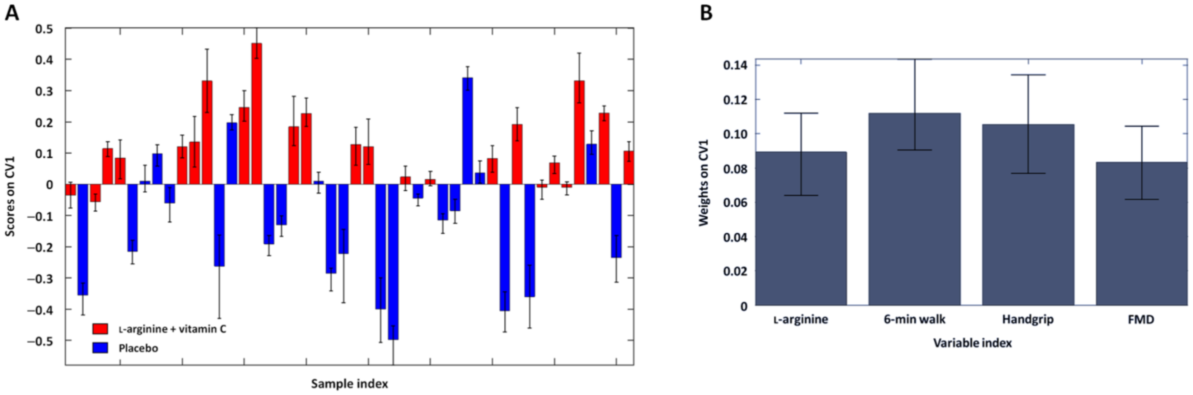

Figure S1. Outer loop sample scores (**A**) and variable weights (**B**) along the only canonical variate of the partial least squares discriminant analysis model. Abbreviation: FMD, flow-mediated dilation

The plots in Figure S1 suggest that for participants treated with L-arginine plus vitamin C there is a more positive variation in the values of the four variables with compared with the placebo group.

In order to rule out a possible effect of age as a covariate, the analysis was repeated by including age as a fifth predictor. The corresponding model yielded an average classification accuracy of 75.5 ± 2.2% and a correct classification rate of 80.7 ± 3.7% in the active group and 70.3 ± 1.9% in the placebo group. These values are lower, though the difference is not statistically significant, than those obtained without including age, suggesting that the observed discrimination is not affected by this covariate. This consideration is strengthened when observing the variable weights along the canonical variate for this second model (Figure S2).

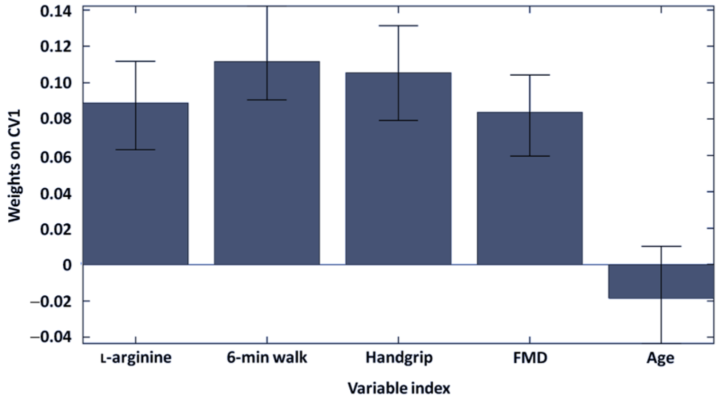

Figure S2. Variable weights along the only canonical variate of the partial least squares discriminant analysis model built including age as a an additional covariate. Abbreviation: FMD, flow-mediated dilation

By looking at the figure, it is evident that the contribution of age as a covariate to the model is not significant, since the 95% confidence interval of its model weight is across zero.

Lastly, to evaluate the effect of sex, separate PLS–DA models were built and validated for males and females. The classification model built on female participants resulted in an average 81.6 ± 3.1% discrimination, corresponding to 83.6 ± 5.4% and 79.6 ± 2.5% correct classification rate for the active and the placebo group, respectively. Also in this case, the figures of merit were all found to be statistically significant ($p < 0.001$) by permutation testing. The model built on male participants yielded an average 55.8 ± 5.3% discrimination, corresponding to 60.0 ± 7.6% and 51.5 ± 7.4% correct classification rate for the active and the placebo group, respectively. When considering the results of permutation test, the discrimination appeared to be not significant ($p=0.23$). However, these last outcomes may be due to the relatively low number of men in the cohort, considering that most male participants were correctly classified in the full model.
